# The complete mitochondrial genome of red-tailed laughingthrush (*Garrulax milnei*)

**DOI:** 10.1080/23802359.2018.1501288

**Published:** 2018-08-27

**Authors:** Lei Zhang, Dajie Xu, Tian Xia, Xiufeng Yang, Guolei Sun, Qinguo Wei, Weilai Sha, Honghai Zhang

**Affiliations:** College of Life Science, Qufu Normal University, Qufu, China

**Keywords:** *Garrulax milnei*, mitochondrial genome, phylogenetic analysis

## Abstract

In this study, the complete mitochondrial genome of *Garrulax milnei* was sequenced for the first time. This mitogenome is 17,871bp in length and it contains 22 tRNA genes, 13 protein-coding genes, 2 rRNA genes, 1 control region and 1 extra pseudo-control region. The base composition of the mitochondrial genome is 29.7% A, 24.5% T, 14.2% G and 31.6% C. The phylogenetic analysis based on the 12 protein-coding genes (except for ND6 gene) showed that *G. milnei* clusters with other two species that belong to genus *Garrulax*.

The *Garrulax milnei*, also called *Trochalopteron milnei*, is classified under the order Passeriformes, family Leiotrichidae (BirdLife International 2016). In this study, the muscle sample of *G. milnei* was collected through the field investigation in Guangzhou province, China, and the geo-spatial coordinates are 23°09′ 52″N latitude, and 1133°30′ 40″E longitude. The samples are deposited in the Herbarium of the Institute of Protection and Utilization for Biological Resource at Qufu Normal University in Shandong province, China. The sample died naturally and DNA was extracted with the DNeasy Blood & Tissue kit (QIAGEN, U.S.A) according to the manufacturer’s protocol. After assembled and annotated, this sequence has been deposited in GenBank with the accession No. MH238447.

The complete mitochondrial genome of *G. milnei* is 17,871 bp in length and encodes 37 genes which contains 22 transfer RNA genes (tRNA), 13 protein-coding genes (PCGs), and 2 ribosomal RNA genes (rRNA). Among these genes, 9 genes (ND6,tRNA*^Ala^*, tRNA*^Asn^*, tRNA*^Cys^*, tRNA*^Gln^*, tRNA*^Glu^*, tRNA*^Pro^*, tRNA*^Ser^*, tRNA*^Tyr^*) encoded in L-strand, and other genes encoded in H-strand. The base composition is 29.7% for A, 24.5% for T, 14.2% for G, 31.6% for C and the percentage of A and T (54.2%) is higher than that of G and C (45.8%). The gene structure, content, and arrangement are similar to other *Garrulax* sequenced in the early research (Huang and Zeng 2016).

Phylogenetic relationships of *G. milnei* and 13 other species were analyzed with Bayesian inference (BI) methods based on 12 protein-coding genes except for *ND6*, and the *Accipiter gentilis* (NC011818) was chosen as an out-group. According to the AIC test criterion, GTR + I + G was selected as the optimal evolutionary model (Tracey et al. 2007) by MrBayes 3.1.2 (Ronquist and Huelsenbeck 2003) and the posterior probabilities are shown on the nodes of the tree. The result of phylogenetic tree showed that *G. milnei* clusters with other two species that belong to *Garrulax (Figure 1)*. To date, few studies have been recorded for *Garrulax*, and we hope that our study can provide a useful database for further research.

**Figure 1.**
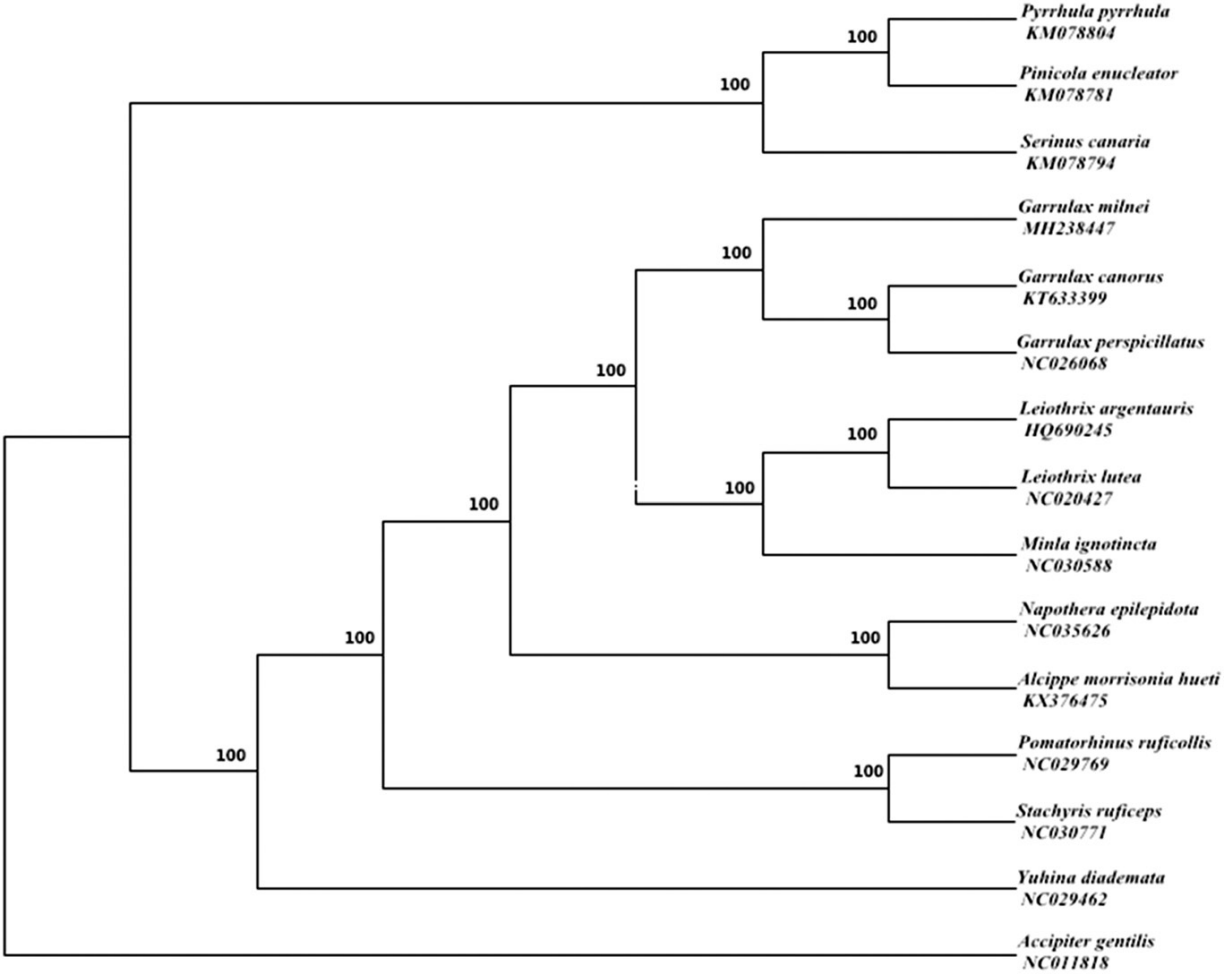
Bayesian phylogenetic inference (BI) trees of 14 species based on 12 protein-coding genes except for *ND6* and the posterior probabilities are shown on the nodes. The accession numbers of 14 species are KM078804 (*P.pyrrhula*), KM078781 (*P.enucleator*), KM078794 (*S.canaria*), KT633399 (*G.canorus*), NC026068 (*G.perspicillatus*), HQ690245 (*L. argentauris*), NC020427 (*L.lutea*), NC030588 (*M.ignotincta*), NC035626 (*N.epilepidota*), KX376475 (*A.M.hueti*), NC029769 (*P.ruficollis*), NC030771 (*S.ruficeps*), NC029462 (*Y.diademata*), NC011818 (*A.gentilis*).
